# A systems model of alternating theta sweeps via firing rate adaptation

**DOI:** 10.1016/j.cub.2024.08.059

**Published:** 2025-02-10

**Authors:** Zilong Ji, Tianhao Chu, Si Wu, Neil Burgess

**Affiliations:** 1 UCL Institute of Cognitive Neuroscience, University College London, 17 Queen Square, London WC1N 3AZ, UK; 2 UCL Queen Square Institute of Neurology, University College London, Queen Square, London WC1N 3BG, UK; 3 School of Psychological and Cognitive Sciences, Beijing Key Laboratory of Behavior and Mental Health, IDG/McGovern Institute for Brain Research, Center of Quantitative Biology, Peking-Tsinghua Center for Life Sciences, Peking University, Haidian District, Beijing 100871, China

## Abstract

Place and grid cells provide a neural system for self-location and tend to fire in sequences within each cycle of the hippocampal theta rhythm when rodents run on a linear track. These sequences correspond to the decoded location of the animal sweeping forward from its current location (“theta sweeps”). However, recent findings in open-field environments show alternating left-right theta sweeps and propose a circuit for their generation. Here, we present a computational model of this circuit, comprising theta-modulated head-direction cells, conjunctive grid × direction cells, and pure grid cells, based on continuous attractor dynamics, firing rate adaptation, and modulation by the medial-septal theta rhythm. Due to firing rate adaptation, the head-direction ring attractor exhibits left-right sweeps coding for internal direction, providing an input to the grid cell attractor network shifted along the internal direction, via an intermediate layer of conjunctive grid × direction cells, producing left-right sweeps of position by grid cells. Our model explains the empirical findings, including the alignment of internal position and direction sweeps and the dependence of sweep length on grid spacing. It makes predictions for theta-modulated head-direction cells, including relationships between theta phase precession during turning, theta skipping, anticipatory firing, and directional tuning width, several of which we verify in experimental data from anteroventral thalamus. The model also predicts relationships between position and direction sweeps, running speed, and dorsal-ventral location within the entorhinal cortex. Overall, a simple intrinsic mechanism explains the complex theta dynamics of an internal direction signal within the hippocampal formation, with testable predictions.

## Introduction

The entorhinal-hippocampal system plays a crucial role in spatial navigation and goal-directed planning.^1–3^ Various spatially tuned cells, including head direction (HD),^4^ place,^5^ grid,^6^ and boundary vector cells,^7^ support the underlying computational processes involved in these functions. Beyond spatial coding, place and grid cells exhibit a temporal coding feature, i.e., theta phase precession, where the sequential firing of individual neurons occurs progressively earlier in relation to the phase of the ongoing theta rhythm driven by inputs from the medial septum.^8,9^ At the populational level, phase-precessing cells underlie forward-directed theta sweeps in linear track environments,^10^ alternating sweeps in T-maze environments^11,12^ and left-right-alternating sweeps in open-field environments.^13^

**Figure 1:**
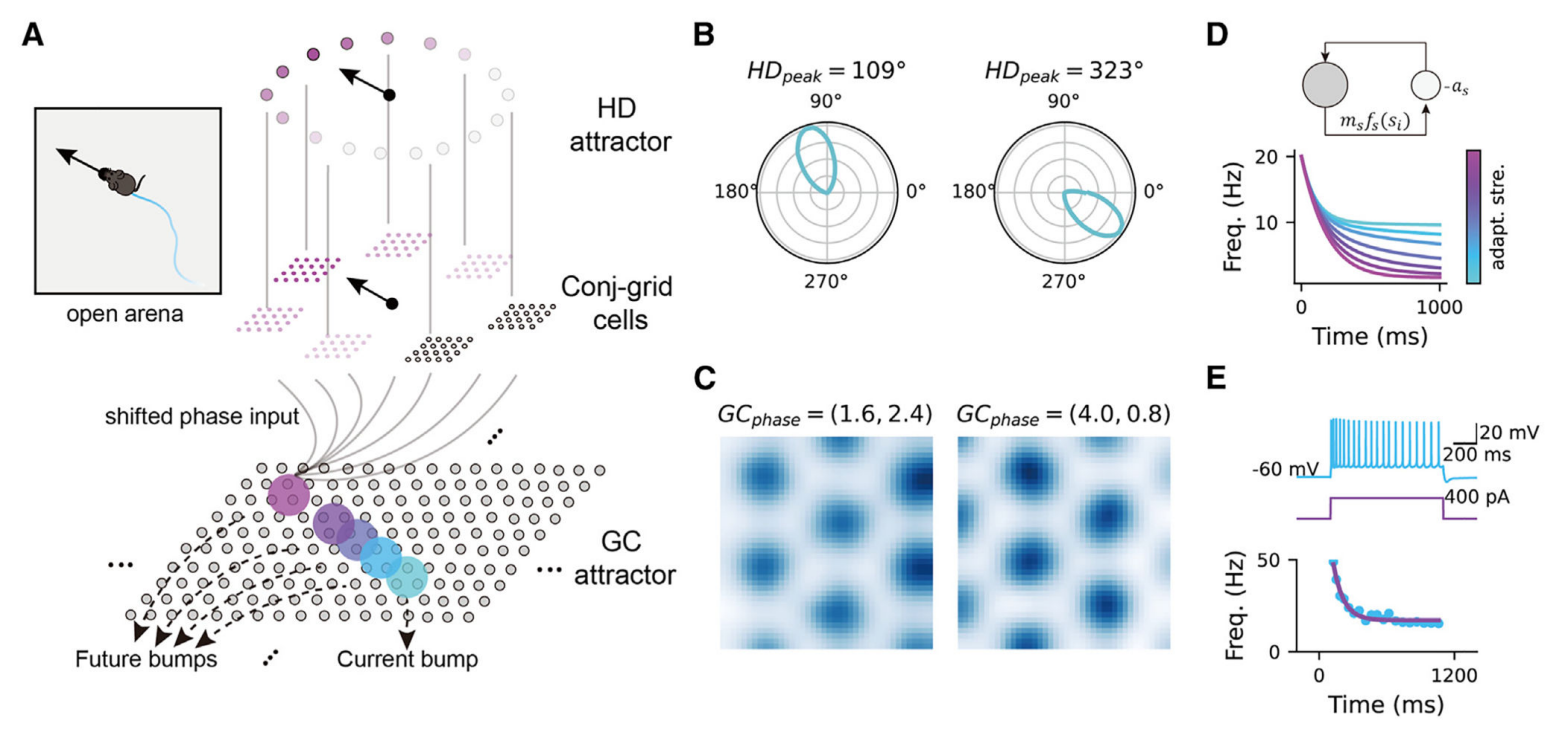
The continuous attractor network model with firing rate adaptation (A) Illustration of the computation model, consisting of a head-direction ring attractor (HD attractor) and a grid cell two-dimensional attractor (GC attractor). The HD attractor receives input of the animal’s head direction, projecting directional input to the conjunctive head × grid cells, which further send a shifted phase input along the direction of head axis to drive the activity bump in the GC attractor. Two example head-direction cells in the HD attractor. (B) Two example grid cells in the GC attractor. (C) Top: illustration of the firing rate adaptation, which is a slow negative feedback modulation to cell firing. Bottom: cell firing frequency as a function of time when applying a constant input current. Different colors represent different adaptation strength. (D) Train (blue) of action potentials elicited in an *in vitro* whole-cell patch-clamp MEC cell during 1-s pulse injection (purple). Instantaneous firing frequency (blue dots) was shown at the bottom with an exponential fit (purple curves). The plot is adapted from Yoshida et al.^21^ with permission. See also Figure S1.

While many network models have been proposed to explain the generation of theta sweeps,^14–16^ they primarily model forward-directed sweeps along the running direction of the animal in linear track environments. This is because the cells are arranged in a one-dimensional manner to fit the environmental setting. It remains unclear how these models can be extended to account for theta sweeps in less constrained environments, such as open fields (but see Chu et al.^17^ for theta sweeps in T-mazes). Inspired by a recent study demonstrating left-right-alternating theta sweeps from recordings of medial entorhinal cortex (MEC) grid cells in open-field environments,^13^ we developed a continuous attractor network model to illustrate theta sweeps in such environments. Our model comprises a three-layer network of theta-modulated HD cells, conjunctive grid × direction cells (hereafter referred to as conj-grid cells), and pure grid cells (Figure 1A). The theta-modulated HD cells,^13,18,19,20^ different from the classic HD cells,^4^ form a ring attractor (hereafter referred to as the HD attractor for simplicity) where the population activity represents an internal direction signal that can differ significantly from the animal’s HD. This HD attractor activates downstream conj-grid cells whose preferred internal directional tuning aligns with the internal direction coded by HD cells. The conj-grid cells then carry position-dependent input with an offset along the internal direction onto the pure grid cells attractor network (hereafter referred to as the GC attractor), driving the activity bump in the GC attractor to move. Due to firing rate adaptation and medial-septal theta modulation, the internal direction represented by the activity bump in the HD attractor sweeps from side to side of the animal’s head axis at theta rhythm. This consequently results in left-right-alternating bump sweeps in the GC attractor via the conj-grid cells.

This model explains many of the empirical findings reported in Vollan et al.,^13^ including (1) the alignment of internal direction and internal location sweeps in separate attractor networks, (2) the linear relationship between sweep length and grid spacing, and (3) the increase of the sweep alternation score with running speed. It also explains and extend many of the empirical findings reported in HD cells,^19,22^ including (1) the relationship between theta sweeps in HD cells at the population level and theta-cycle skipping at the single-cell level and (2) broader tuning width of HD cells exhibiting theta skipping than those which do not. Moreover, we make several new predictions. These include how the modulatory theta input affects theta sweeps in both the HD and the grid cell networks, how the HD network controls theta sweeps in the downstream grid cell network, how animals’ behaviors (e.g., running speed, straight runs, and turns) modulate theta sweep features, and how theta sweeps vary in different grid modules along the MEC dorsal-ventral axis. Our model also predicts theta-modulated HD cells with specific properties, which we test with empirical data from theta-modulated HD cells recorded in the anteroventral thalamic nucleus.^19^

In summary, our model proposes a mechanism for intrinsic generation of theta sweeps in hardwired neural networks within the brain’s spatial navigation system. This model enhances our understanding of the generation and nature of theta sequences, potentially shedding light on the neural dynamics underlying various navigational behaviors.

## Results

### The computational model

The computational framework, following the neural circuit outlined in Vollan et al.,^13^ is composed of coupled continuous attractor networks modeling theta-modulated HD cells in the parasubiculum as a ring attractor (HD attractor) and grid cells in the MEC as a two-dimensional attractor on a neuronal sheet (GC attractor) (see Figures 1A–1C and S1 and STAR Methods for details).

Note that neurons in the HD attractor differ from classic (non-theta-modulated) HD cells.^4^ Instead, they encode an internal direction signal that differs from the animal’s HD—for example, by alternating left and right of the head axis—and may encode something fundamentally different, such as intended movement direction. When the animal runs in the environment, HD-dependent sensory input activates an activity bump in the theta-modulated HD attractor, whose center on the ring represents the internal direction. Activated theta-modulated HD cells that code for the internal direction (referred to as HD cells hereafter for simplicity) then activate downstream conj-grid cells in MEC layer 3, which share the same preferred internal direction as those activated HD cells. The conj-grid cells further transmit a position signal as a phase input onto the GC attractor manifold, with the input strength modulated by the animal’s running speed.^23^ Importantly, this phase input is slightly shifted along the preferred internal direction of the activated conj-grid cells,^13^ driving the activity bump in the GC attractor (representing the internal location of the animal) to move in that direction.

Neurons in the model exhibit firing rate adaptation. At the single-neuron level, this leads to a reduction in a neuron’s firing rate in response to an input of constant intensity (Figures 1D and 1E). At the network level, it induces intrinsic mobility of the activity bump by destabilizing it.^24^ This intrinsic mobility interacts with the external drive of sensory input, resulting in sweeps of the activity bump^17^ (see below). Furthermore, both the HD cells and the grid cells in the model receive theta rhythmic input from the medial septum^25^ with input strength increasing with the animal’s running speed (STAR Methods). This theta input governs the sweep frequency of the activity bumps induced by firing rate adaptation. In summary, the combination of intrinsic firing rate adaptation and external theta modulation results in internal direction sweeps in the HD network, which further drives the internal location sweeps in the GC attractor network via an intermediate layer of conj-grid cells (STAR Methods).

### Bidirectional and forward sweeps in theta-modulated HD cells

**Figure 2:**
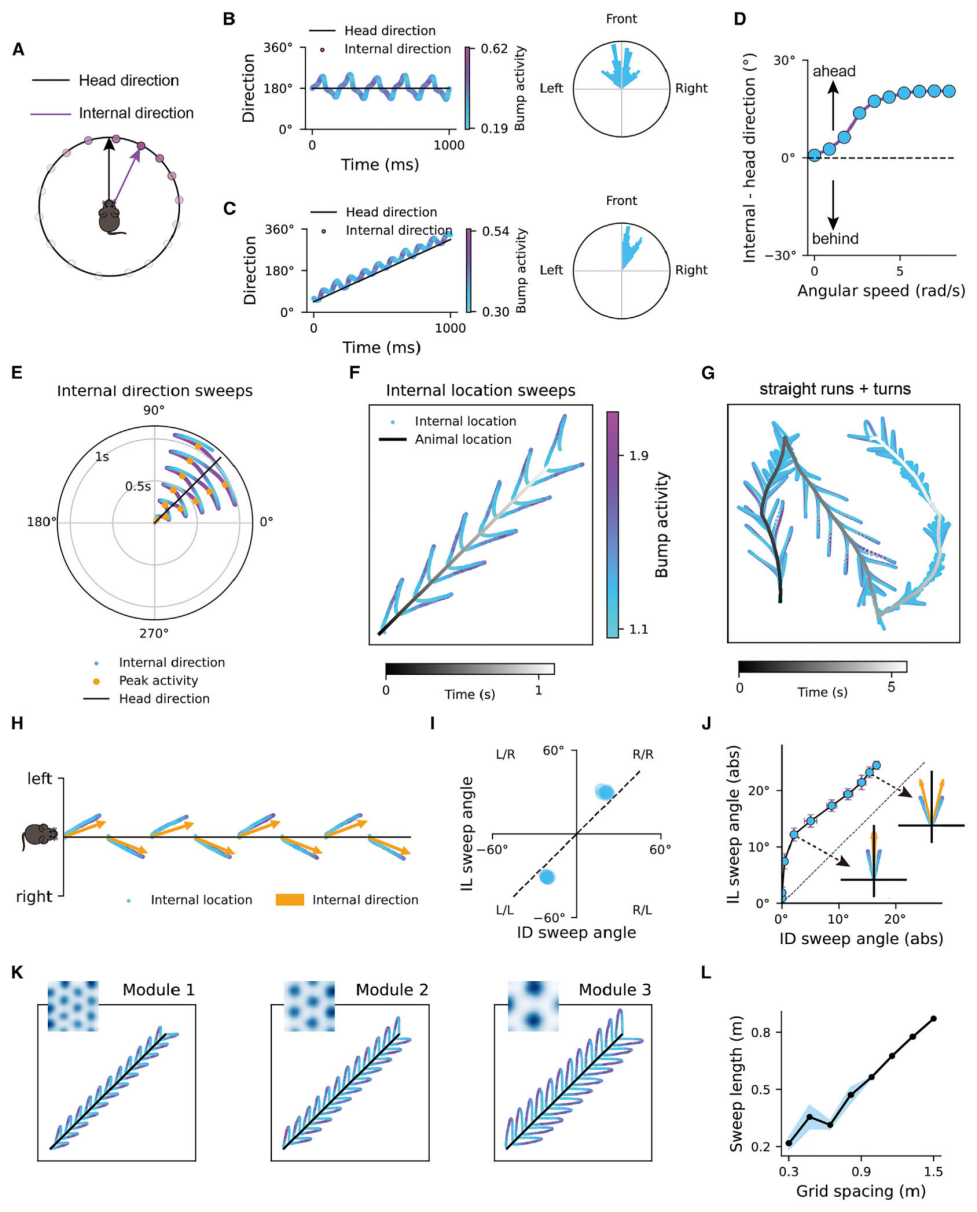
Alternating internal direction sweeps in HD cells and internal location sweeps in grid cells (A) A schematic of head direction and internal direction represented by the activity bump in the HD attractor. (B) Left: bidirectional theta sweeps when the animal runs along a straight trajectory with fixed head direction (the dark line). Blue to purple dots represent the internal direction (the bump center) across 1-s simulation. More purple dots represent higher peak firing rate of the activity bump. Right: polar histogram of the offset angle of the internal direction relative to the head axis over time. Note that the cluster around the peak of internal direction may occur because the activity bump requires some time to change direction, during which its speed reduces to zero before increasing again. (C) Left: forward-directed theta sweeps when the animal turns right (clockwise) with a constant angular speed. Right: polar histogram of the offset angle of the internal direction relative to the head axis over time. (D) The averaged offset of the internal direction relative to the head axis as a function of head rotation speed. Positive values represent the internal direction leading ahead of the head direction. (E) Internal direction sweeps in the model when the animal runs along a straight line with a fixed head direction (45°). More purple dots represent higher firing rate of HD cell population. (F) Internal location sweeps from the pure grid cells, with the gray line representing the animal’s trajectory over time and the dots representing the internal location. More purple dots indicate higher firing rate of grid cell population. (G) Internal location sweeps on a trajectory, including straight runs, turns, and immobile periods (see also Video S1). (H) The internal location sweeps in grid cells align with the internal direction sweeps in upstream head-direction cells. (I) A scatterplot of the internal direction sweep angle and the internal location sweep angle across all theta cycles on a simulated straight run. The gray dashed line represents identical value of the two sweep angles. (J) The internal location sweep angle increases with the internal direction sweep angle, with the insets showing two example sweeps with different angles. The dashed line marks the identical sweep angles of the internal direction and the internal location. (K) Internal location sweeps in grid modules with different grid spacing. (L) Location sweep length is proportional to grid spacing. Dots represent the mean of 10 simulations at each grid spacing, with the shaded area representing the standard deviation. See also Figures S2–S4 and Videos S1 and S2.

We define the center of the activity bump on the theta-modulated HD attractor manifold as the internal direction of the animal^13^ (Figure 2A). From the perspective of population activity, the internal direction is a representation decoded from the collective activity of HD cells. This internal direction can differ from the actual HD in theta-modulated HD cells, unlike in classic, non-theta-modulated HD cells. We first demonstrate that with firing rate adaptation, theta-modulated HD cells exhibit sweeps of internal direction. Depending on whether the animal rotates its head, the internal direction exhibits either bidirectional sweeps (side to side of the head axis) or forward sweeps (of angle in the direction of rotation). Specifically, when the animal runs along a straight trajectory with a fixed HD, the internal direction exhibits bidirectional sweeps from side to side of the head axis (Figure 2B), with the averaged offset between internal direction and HD equal to zero (Figure 2D). This aligns with the discrete, alternating activity observed from side to side of the head axis in HD cells during fast and straight running.^13^

Furthermore, when the animal’s head rotates, the internal direction exhibits forward sweeps of angle in the direction of rotation during turning (Figure 2C). This ahead representation explains previous findings related to anticipatory firing in HD cells.^26,27^ Importantly, the forward theta sweeps predict the existence of theta phase precession for turning angle in theta-modulated HD cells (see results below). Moreover, we reveal a continuous spectrum from bidirectional sweeps to forward sweeps as the angular speed increases. Specifically, as the animal rotates its head faster, internal direction sweeps become more ahead of the direction of rotation in a linear manner (Figure 2D). While alternating activity in HD cells is most significantly observed during fast straight runs in open-field environments,^13^ forward sweeps during turning periods are an important contribution to the overall pattern of results (Figure S2; Video S1).

### Internal direction sweeps in HD cells drive alternating location sweeps in grid cells

Here, we demonstrate that when the animal runs in a straight line, the bidirectional sweeps of internal direction, combined with firing rate adaptation in the grid cell network, drive left-right-alternating sweeps of internal location in grid cells (Figures 2E and 2F). Moreover, since the effect of firing rate adaptation in the HD network varies with turning and hence affects the dynamics in downstream grid cells, we also demonstrate theta sweeps in a segment of the real trajectory of a rat, including straight runs, turns, and immobile periods (see Figure 2G and Video S1). For straight runs, the sweep angle of internal location (defined as the angle between location sweep trajectory and the movement trajectory) is 24.6° ± 1.3°, while the sweep angle of internal direction (defined as the peak offsets, i.e., the direction offsets when HD cells arrive at the peak firing rate, on either side of the head axis) is 17.0° ± 0.7° (Figure 2D), which is comparable to empirical data^13^ (see Table S1 for parameter settings). Importantly, the internal direction sweep in the upstream HD cells drives the internal location sweep, directing it to the same side of the head axis across theta cycles (see Figures 2H and 2I and Vollan et al.^13^). Moreover, the sweep angle of the internal direction contributes to the sweep angle of the internal location (Figure 2J).

Different grid modules form independent continuous attractor networks^13^ and sweep length was found to increase proportionally with the grid spacing in each module.^13^ Our model implemented this finding by adjusting the ratio of mapping between the physical space and the phase space defined by the toroidal unit (STAR Methods). An increased mapping ratio results in larger grid spacing (Figure 2K). While different attractor networks receive the same speed input in physical space (the animal’s speed), networks with larger grid spacing have proportionally lower speed input in phase space after transformation. Importantly, the speed variation in phase space has only a minor effect on the sweep length on the toroidal manifold (Figure S3), indicating that the sweep lengths are equal across grid modules when mapped onto the module’s toroidal unit tile.^13^ The invariance of sweep length across modules, when mapped back to physical space, in turn increases proportionally with the mapping ratio, thus correlating linearly with the grid spacing (see Figures 2K and 2L and Video S2).

Instead of teleporting instantaneously back to the animal’s location at the end of each theta cycle, the activity bump sweeps continuously back to the animal’s location, because of the continuous nature of the GC attractor. During outward sweeps, grid cells exhibit higher firing rates than during inward sweeps (see Figures 2F, 2K, and S4), because the firing of cells on the outward sweep path causes firing rate adaptation, reducing their firing rate when the activity bump sweeps inward. We found the same pattern in experimental data from grid cells (Video S3), notwithstanding the presentation in Vollan et al.,^13^ which emphasizes the forward sweeps.

### Speed modulation of theta sweep features

**Figure 3:**
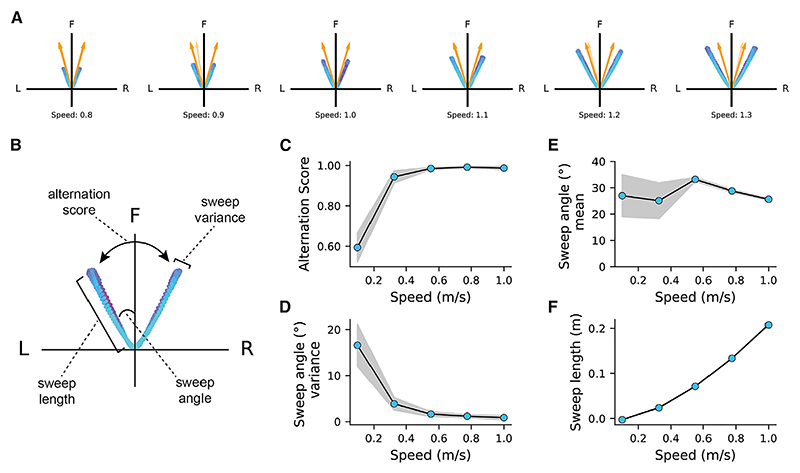
Speed modulation of sweep features (A) Left-right-alternating theta sweeps of internal direction in the HD attractor (orange arrows) and internal location in the GC attractor (blue-purple dots) under different running speeds. (B) Schematic of extracting the mean sweep angle, the variance of sweep angle, the sweep length, and alternation score. (C) The relationship between the alternation score of location sweeps in grid cells and running speed of the animal. Shaded area represents the standard deviation of the results from 10 simulations of the model under each running speed. (D) The relationship between the variance of sweep angle and running speed. (E) The relationship between the mean sweep angle and running speed. (F) The relationship between the sweep length and running speed.

In our model, running speed increases both the strength of medial-septal theta modulation (consistent with experimental data^28^) and the strength of shifted phase input from the conjgrid cells to the GC attractor network.^23^ These two factors jointly affect both the sweep features in the HD attractor network and the downstream GC attractor network (Figure 3A). We checked four sweep features, including the alternation score (STAR Methods), the mean sweep angle, the variance of the sweep angle, and the internal location sweep length (Figure 3B). We first verified that the alternation score of theta sweeps in the GC attractor network increases with running speed (Figure 3C), while the variance of sweep angle decreases with running speed (Figure 3D). These two results agree with the empirical observation of increased sweep bimodality with running speed.^13^

We made additional testable predictions related to other sweep features. First, the mean sweep angle remains roughly the same as running speed increases (Figure 3E), indicating that the sweep angle is minimally affected by running speed. Second, the sweep length increases almost linearly as running speed increases (Figure 3F). This is also due to stronger input from conj-grid cells at higher speed,^23^ which drives the activity bump to move further. If hippocampal place cell sweeps are inherited from entorhinal grid cells,^13^ this could also explain the linear relationship of theta sweeps and speed found in the hippocampus.^29^

### Firing rate adaptation modulates theta sweeps along the MEC dorsal-ventral axis

**Figure 4:**
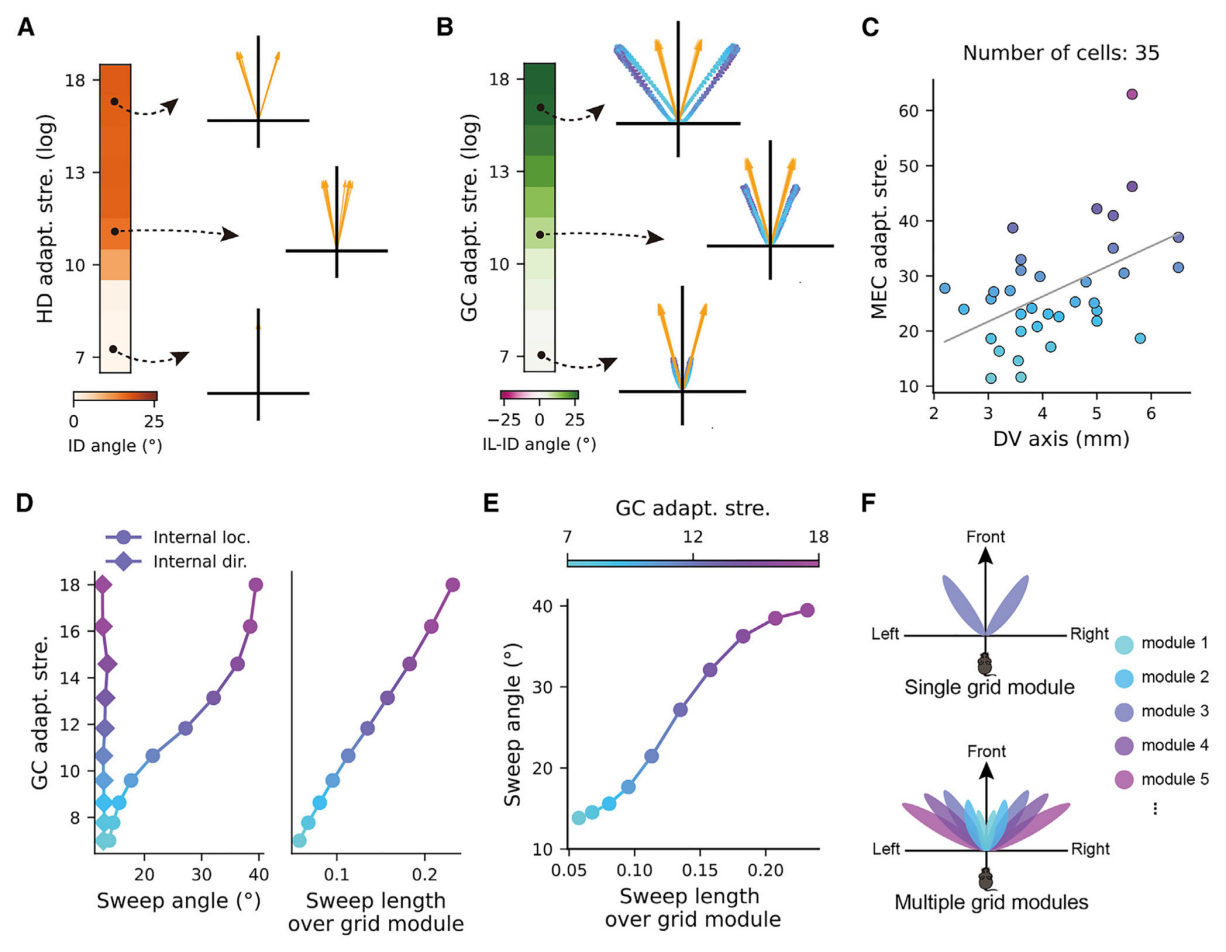
Adaptation strength modulates theta sweeps along the MEC dorsal-ventral axis (A) The sweep angle of internal direction increases with the strength of firing rate adaptation in head-direction cells, with three examples shown on the right. (B) The angular difference between the location sweep and the direction sweep increases with the strength of firing rate adaptation in grid cells (while the adaptation strength in head-direction cells is fixed), with three examples shown on the right. Green/pink color represents larger/smaller sweep angle in internal location than that in internal direction. Note that the angular difference is always positive, meaning that the location sweep angle is larger than the direction sweep angle. (C) The adaptation strength as a function of locations along the dorsal-ventral axis for 35 cells in the MEC with *in vitro* whole-cell patch-clamp recording. The gray line shows a linear fit (Pearson correlation with r = 0.48, *p* = 0.003). The data was obtained from Yoshida et al.^21^ with permission. (D) Left: the sweep angles as a function of increased adaptation strength in the GC attractor network (with the adaptation strength in the HD attractor network fixed). Right: same as the left but shows the sweep length of internal location (normalized by the effect of grid spacing) as a function of increased adaptation strength along the dorsal-ventral axis. (E) The sweep angles correlate positively with the sweep lengths (normalized by the effect of grid spacing) while increasing the adaptation strength in grid cells along the dorsal-ventral axis. (F) Schematic of sampling of surrounding space with a single grid module versus with multiple grid modules. More purple colors represent sweeps in grid modules with larger adaptation strength and larger grid spacing. See also Figure S5.

In the extreme case, without any firing rate adaptation, the internal direction tracks the animal’s HD, while the internal location tracks the animal’s position (Figure S5). In the more general case, as adaptation strength increases, our model generates several predictions that can be tested in future experiments. First, increasing the adaptation strength in HD cells increases the theta sweep angle of the internal direction (Figure 4A). Second, increasing the adaptation strength in grid cells increases the theta sweep angle of the internal location (Figure 4B). Furthermore, while sweeps in grid cells are driven by sweeps in HD cells, the location sweep angle is larger than the direction sweep angle, due to the additional firing rate adaptation in grid cells (see Figure 4B and also Figures 2J and 3A). Increasing the adaptation strength in grid cells increases the angular difference between the location sweep and the direction sweep (Figure 4B).

While evidence for changes of adaptation strength across HD cells is lacking, the strength of firing rate adaptation increases along the MEC dorsal-ventral axis^21^ (Figure 4C). This leads to a third prediction, which is that the location sweep angle is larger in grid modules located in the more ventral areas of the MEC (Figure 4D). Moreover, stronger adaptation also leads to an increase in the sweep length of internal location, even when expressed as a proportion of grid scale (Figure 4D). This suggests a positive correlation between sweep angle and sweep length across multiple grid modules along the dorsal-ventral axis because of the increase in adaptation strength (Figure 4E); that is, shorter sweeps in dorsal grid modules with smaller grid spacing are more forward directed, while longer sweeps in ventral grid modules with larger grid spacing have larger sweep angles. When the animal runs in an open field, theta sweeps from multiple grid modules can therefore cover a much larger surrounding space than single-module theta sweeps, potentially contributing to efficient sampling of the surrounding space without the need for physically visiting it (Figure 4F).

### Theta phase precession correlates with theta skipping in HD cells

**Figure 5:**
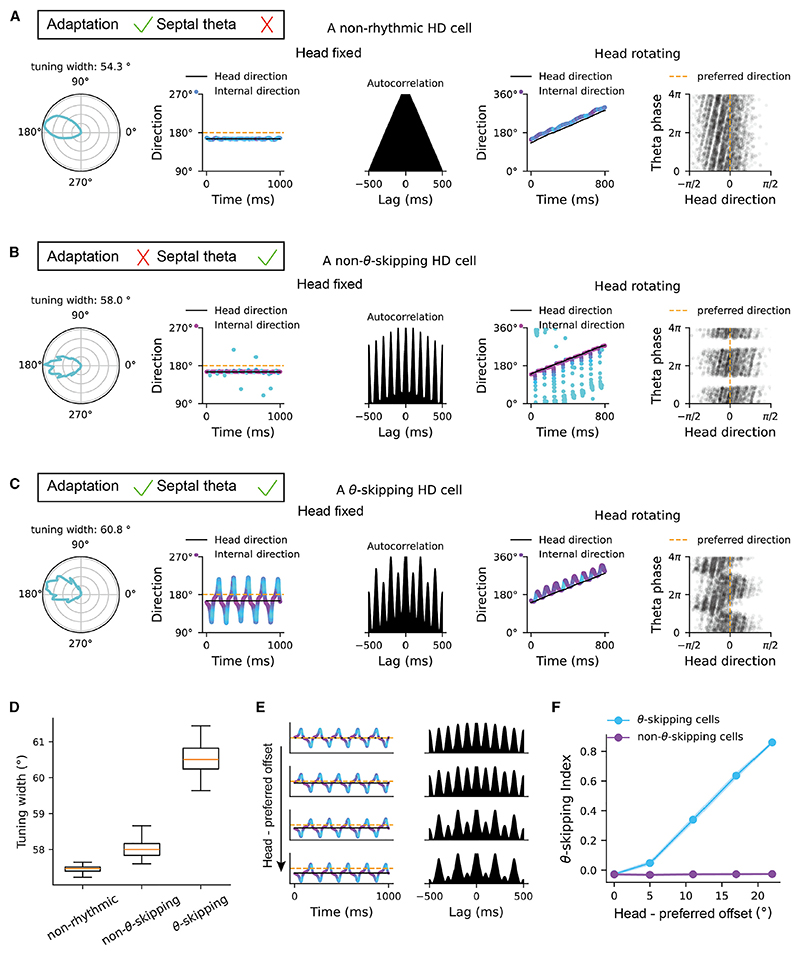
Theta skipping and theta phase precession in the head-direction ring attractor (A) A classic HD cell without theta modulation, simulated with a HD attractor with firing rate adaptation but without septal theta input. From left to right: the tuning field of a cell; the internal direction over 1-s time window when the animal’s head is fixed, with orange line representing the preferred direction of a probed cell; the auto-correlation of the firing of the probed cell; the internal direction over 1-s time window when the animal’s head is rotating with a constant angular speed; the spike phase of the probed cell as a function of head direction. (B) A HD cell with theta modulation but without theta skipping, simulated with septal theta input but without firing rate adaptation. (C) A HD cell with theta skipping, simulated with both septal theta input and firing rate adaptation. (D) The tuning width of the three types of HD cells. (E) Increased theta-skipping effect as the fixed head direction (dark lines) is more away from the preferred direction of a probed cell (orange lines). (F) Theta-skipping index increases as a function of the offset between the actual head direction of the animal and the preferred internal direction of a cell for theta-skipping HD cells (blue), but not for normal theta-modulated HD cells without skipping (purple).

**Figure 6:**
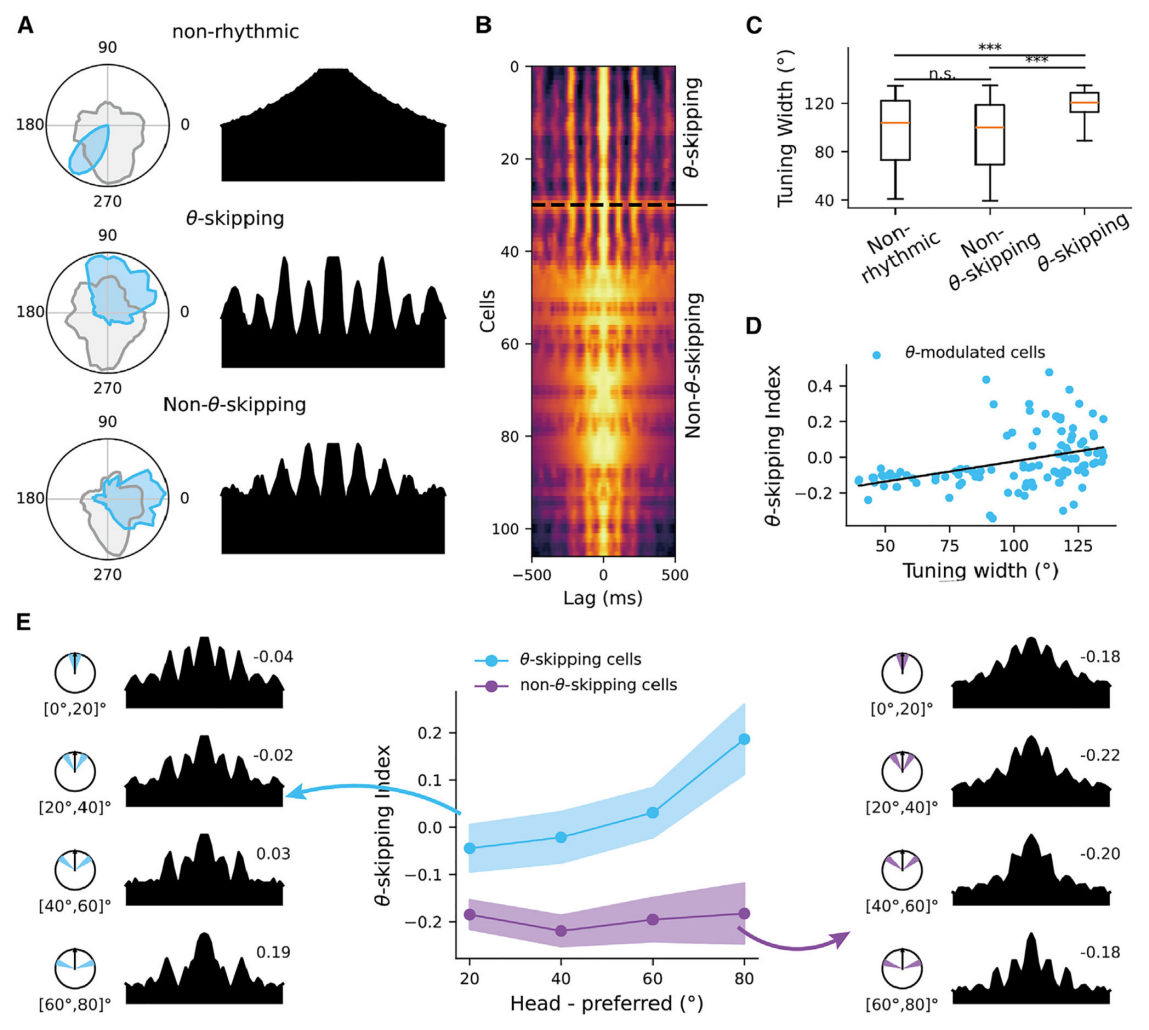
Theta skipping in the HD cells in the anteroventral thalamic nucleus (A) Examples of a classic HD cell (non-rhythmic), a normal theta-tuned HD cell without skipping (non-theta-skipping), and a theta-skipping HD cell. Left column: the tuning width. Right column: the auto-correlation. (B) Heatmap of auto-correlations of all theta-modulated HD cells, ordered by theta-skipping index. Warmer colors represent higher correlation values. Top rows are for theta-skipping cells, and bottom rows are for normal theta-tuned HD cells without skipping. (C) The tuning width of the three types of HD cells (classic vs. non-theta-skipping: [MWUT] with Z = 1.0, *p* = 0.306; classic vs. non-theta-skipping: [MWUT] with Z = 3.8, *p* = 1.5×10^-4^; classic vs theta-skipping [MWUT] with Z = 4.6, *p* = 5.1×10^-6^)). (D) Theta-skipping index and tuning width for theta-modulated HD cells are positively correlated (Pearson correlation with r = 0.41, *p* = 1.53 × 10^−5^). Each dot is a cell, and the dark line represents a linear fitting. (E) Theta-skipping index as a function of the offset between head direction and the preferred direction for both non-theta-skipping HD cells (purple) and theta-skipping HD cells (blue). The auto-correlation plots were taken by averaging the auto-correlation of each cell in the offset range showed on the disk, with preferred directions all aligned to the north.

While classic HD cells have been reported in many brain regions such as anterodorsal thalamus, lateral mammillary bodies, and retrosplenial cortex, theta-modulated HD cells have also been found recently in the anteroventral thalamic nucleus,^18,19^ parasubiculum, and entorhinal cortex.^13,22^ By varying the theta input and firing rate adaptation, our HD attractor module generates three types of HD cells observed in previous studies (Figures 5A–5C and 6A): specifically, (1) without medial-septal theta input, cells in the HD attractor exhibit the features of classic HD cells (Figure 5A); the activity bump leads ahead of the animal’s HD when rotating, giving rise to anticipatory firing in HD cells^26,27^; (2) with theta input but without firing rate adaptation, cells in the HD attractor exhibit the features of theta-modulated HD cells where HD cells fire in every theta cycle (Figure 5B); and (3) with both theta input and firing rate adaptation, cells in the HD attractor exhibit the features of theta-skipping HD cells where HD cells fire in alternating theta cycles (Figure 5C).^22,30^

From the populational view, theta skipping of individual neurons can originate from the sweeps of activity bumps, which leads to two interesting predictions. First, bump sweeps increase the tuning width of theta-skipping HD cells, compared with classic HD cells, and normal theta-modulated HD cells where there is no bump sweep (Figure 5D). Second, the theta-skipping effect becomes more significant when the animal runs along a direction with a larger offset to the preferred internal direction of the cell, as the activity bump is more likely to sweep over the preferred internal direction in alternating theta cycles (Figures 5E and 5F).

Finally, our HD attractor with firing rate adaptation and medialseptal theta input predicts the existence of theta phase precession in theta-skipping HD cells when the animal turns through the preferred internal direction of the cell (Figure 5C). This corresponds to network models of theta phase precession in place cells when the animal runs in linear track environments,^14,15,17^ where the activity bump sweeps over the preferred location of a place cell at progressively earlier phases of theta cycles when the animal traverses the firing field.

We verified predictions related to the firing features of theta-skipping HD cells, with empirical data containing a larger number of HD cells recorded from the anteroventral thalamic nucleus^19^ (Figures 6A and 6B; number of classic HD cells: 76; normal theta-modulated HD cells: 77; theta-skipping HD cells: 30). First, directional tuning width is significantly larger in theta-skipping HD cells than normal theta-modulated HD cells (Mann-Whitney U test [MWUT] with *Z* = 4.6, *p* = 5.1 × 10^−6^) and classic HD cells (MWUT with *Z* = 3.8, *p* = 1.5 × 10^−4^), while the tuning width in normal theta-modulated HD cells and classic HD cells is similar (MWUT with *Z* = 1.0, *p* = 0.306), which agrees with the model where the activity bump does not sweep in these two cases (Figure 6C). Interestingly, cells exhibiting stronger theta-skipping effect have broader tuning width (Figure 6D; Pearson correlation with r = 0.41, *p* = 1.53 × 10^−5^). Second, for theta-skipping HD cells, the skipping effect increases during the periods when the animal’s HD is more further away from the preferred internal direction of the cell, while for normal theta-modulated HD cells, the skipping effect remains weak and independent of the animal’s HD (Figure 6E).

### Theta sweeps disappear with septal inactivation

Reduction in theta oscillation by pharmacological inactivation of the medial septum correlates with impairment in spatial memory tasks.^31^ At the neurophysiological level, septal inactivation eliminates theta skipping in HD cells in the parasubiculum and MEC,^22^ and it impairs phase precession in the entorhinal cortex and hippocampus.^32,33^ Inspired by these phenomena, we investigated how septal inactivation affects network dynamics in our model.

**Figure 7:**
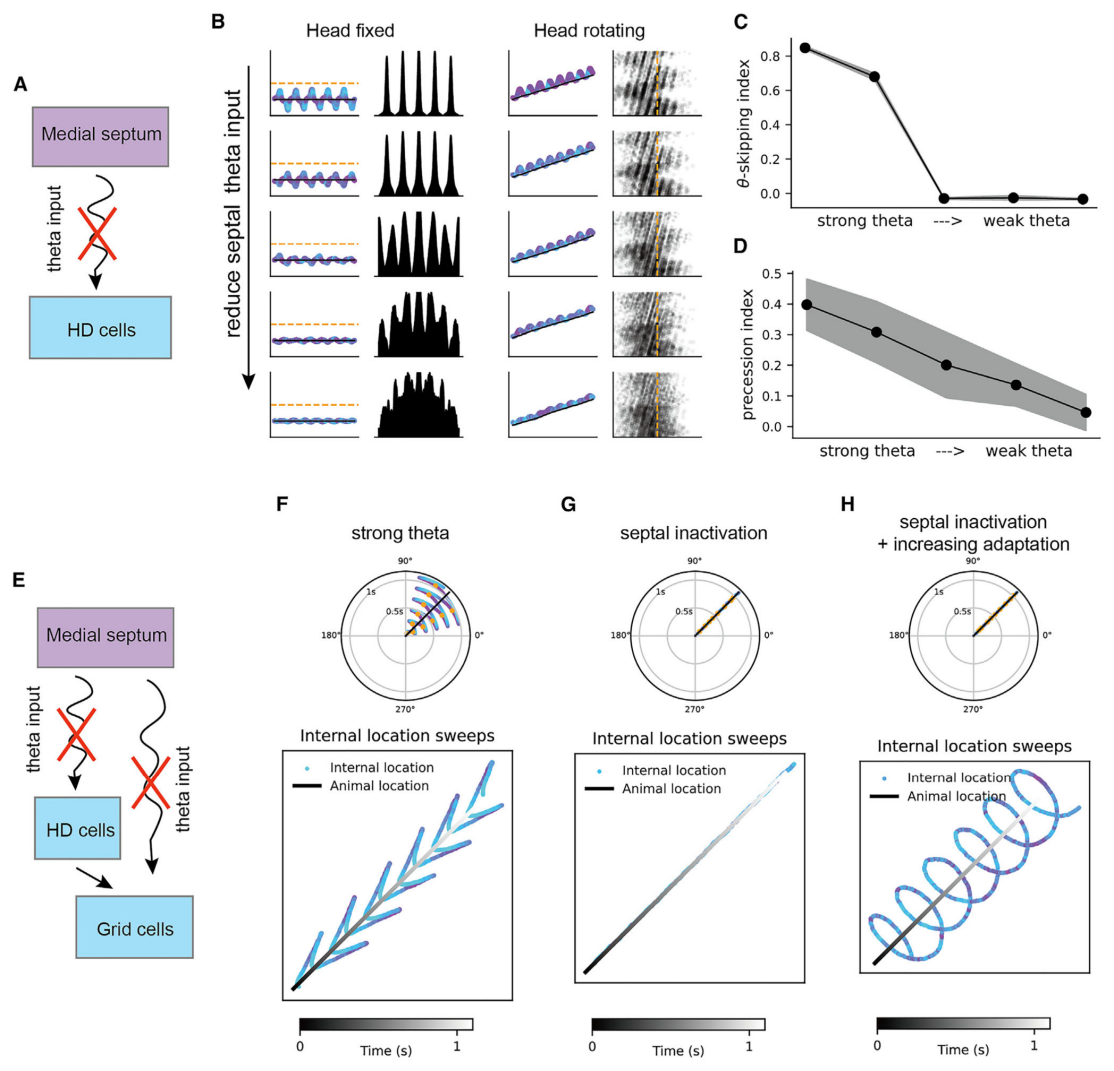
Theta sweeps disappear with septal inactivation (A) Schematic of inactivating medial-septal theta input to the HD cells. (B) Reduced effect of theta skipping with head fixing (left two columns) and theta phase precession with head rotating (right two columns), with weakening of the theta input strength from the medial septum. (C) Theta-skipping index decreases with weakening of the theta input strength. (D) Circular linear correlation coefficient decreases with weakening of the theta input strength. (E) Schematic of inactivating medial-septal theta input to both the HD cells and the grid cells. (F) Left-right sweeps in grid cells with medial-septal theta input. (G) The activity bump tracks the animal’s position without medial-septal theta input. (H) Circular sweeps in grid cells without medial-septal theta input under increased adaptation strength.

First, we showed that in the HD network (Figure 7A), reducing the theta rhythm magnitude decreases the occurrence of direction sweeps and therefore theta skipping in HD cells (Figures 7B and 7C), which aligns with previous empirical results.^22^ Moreover, since theta skipping and phase precession are two aspects of the same network (depending on whether or not the head is rotating), we predicted that reducing the theta modulation magnitude also decreases the effect of theta phase precession relative to the HD angle in HD cells (Figures 7B and 7D).

Second, we showed that in the downstream grid cell network (Figure 7E), inactivating the septal theta rhythm leads to two dynamics different from the left-right sweeps under normal settings of firing rate adaptation and septal theta input (Figure 7F): specifically, (1) with relatively weak adaptation in grid cells, both the internal direction and location track the animal’s HD and position (Figure 7G); and (2) with relatively strong adaptation in grid cells, while the internal direction still tracks the animal’s HD, the internal location exhibits circular sweeps around the animal’s position for 360° within individual theta cycles (Figure 7H). While such circular sweeps have not been reported in empirical data, they might exist as intrinsic network dynamics under weak theta modulation from the medial septum when running speed is low.

These predictions could be tested in future inactivation experiments, bearing in mind that rotational theta may have a cholinergic component,^34,35^ compared with translational theta, which is controlled by GABAergic and glutamatergic medial-septal cells.^36^

## Discussion

We demonstrated that a continuous attractor model with firing rate adaptation and medial-septal theta modulation is sufficient to explain recent experimental findings of left-right-alternating sweeps in grid cells and related directional “flickering” in theta-modulated HD cells.^13^ In this model, the upstream HD network generates internal direction sweeps at the theta rhythm, which activates the conj-grid cells that share the same preferred internal direction as those activated HD cells. These conj-grid cells then provide a shifted input along the same direction, driving the left-right-alternating sweeps in grid cells in a coordinated manner. This model accounts for many of the experimental findings, including the alignment of internal direction sweeps in parasubicular HD cells and internal location sweeps in grid cells, with the former driving the latter; the linear relationship between location sweep length and grid spacing across different grid modules; and the increase of the sweep alternation score with running speed.

The proposed model is improved in three aspects over previous network-based models of theta sweeps and theta phase precession when the animal runs along a linear track.^14–17,37^ First, while phase precession has been observed in both grid cells and place cells in open fields,^38,39^ models for populational theta sweeps extending beyond linear track scenarios have not been well addressed so far. However, recent progress has been made, including our modeling of left-right theta sweeps in place cell networks when the animal runs toward a decision point in a T-maze,^12^ as well as a similar model with firing rate adaptation and theta input, which accounts for left-right-alternating sweeps in place cells in an open field.^40^ Second, we systematically modeled theta sweeps in a system of HD cells and grid cells, with sweep direction in HD cells determining sweep direction in downstream grid cells and place cells.^13^ Instead of generating theta sweeps independently in grid cell networks across multiple grid modules and place cell networks, the coordination of sweep directions across multiple networks by HD cells may help synchronize the temporal organization of spiking activity across multiple brain regions. This synchronization could potentially support the stabilization of a single coherent map for spatial navigation and episodic memory function.^37,41,42^ Third, our model extends beyond fast straight runs in open fields by simulating a variety of running trajectories (Figure 2F), including turning, stopping, accelerating, decelerating, and random walking (Figures 2G and S2). This broader scope can help provide a more comprehensive understanding of theta sweeps and guide experimental data analysis in more complex behavioral scenarios.

The model only requires attractor dynamics, firing rate adaptation, theta rhythmicity, and an external input to generate coherent theta weeps. The external sensory input is not necessarily from actual perception, as virtual trajectories can occur during both rapid eye movement (REM) and slow-wave sleep states. When theta rhythmicity manifests together with the external input, the network will still generate theta sweeps, as seen during REM sleep but not during slow-wave sleep.^13^ Equally, the decoding of theta sweeps occurs on the toroidal manifold of the grid attractor, and it is then mapped onto physical space with a consistency constraint to resolve periodic ambiguity (STAR Methods), which allows decoding of theta sweeps to unvisited locations^13^ (Video S1).

Our modeling and empirical results favor continuous sweeps of direction and location, instead of discrete flickers from side to side of the animal’s head axis in the HD network and forward-only grid cell sweeps as presented by Vollan et al.^13^ These continuous sweeps align with the continuous attractor nature of the HD system^43,44^ and our finding of theta phase precession during turning, which may also affect the continuity of theta sweeps in downstream grid cells. Using data from Gardner et al.,^45^ we decoded theta sweeps from grid cell population activity and found that the decoded position likely sweeps continuously backward to the animal’s location within individual theta cycles (see Figures S6 and Video S3). While our model favors continuous sweeps in grid cells, we did observe higher network activity during outward sweeps, compared with inward sweeps (see Figures 2F, 2K, and S4; also see Tsodyks et al.^14^ and Romani and Tsodyks^15^), which may affect the continuity of decoded position within each cycle. Thus, we consider the continuous sweeps reported here, compared with the left/right flickering of directional sweep and forward-only locational sweeps reported by Vollan et al.,^13^ to reflect a quantitative difference in thresholding for presentation rather than a real qualitative difference.

Our model makes numerous novel predictions related to the features of theta sweeps in both HD cells and grid cells. In theta-modulated HD cells, we predicted (1) increased directional tuning width in theta-skipping cells and (2) increased theta-skipping effect when the offset between the HD and the preferred internal direction increases, both of which we verified with empirical data from theta-modulated HD cells in anteroventral thalamus.^19^ Moreover, our model also predicts the existence of theta phase precession for turning angle in theta-modulated HD cells and specifically those displaying theta skipping, which we will check in detail in future.

In grid cells, we predict the following: (1) as the animal runs faster, the sweep length increases while the variance of the sweep angle decreases; (2) within a single grid module, the sweep angle in grid cells is always larger than that in upstream HD cells; and (3) across multiple grid modules along the MEC dorsal-ventral axis, there is a systematic increase in both the length and angle of theta sweeps. Compared with single-module sweeps, the multi-module sweeps provide an efficient way of covering a surrounding space without physically visiting them, potentially contributing to an efficient formation of new maps for spatial navigation (Figures 4E and 4F).^46–48^ Moreover, we predict reduced effects of theta skipping, phase precession, and theta sweeps when the medial-septal theta rhythm magnitude decreases, which could be tested with septal inactivation in future experiments.

Our model suggests that two temporal firing features in theta-modulated HD cells, i.e., theta skipping and theta phase precession, are two aspects of the same underlying network dynamics of theta sweeps. This is supported by an empirical observation in data recorded from the anteroventral thalamic nucleus^19^: theta skipping HD cells, but not normal theta-modulated HD cells, show an increase in the skipping index as the animal’s HD deviates further from the cells’ preferred internal directions (Figure 6E). Since the classic HD signal is generated subcortically, arriving at the neocortex via the mammillary bodies and anterodorsal thalamus at the dorsal presubiculum,^49^ we hypothesize that theta-modulated HD cells in the parasubiculum inherit their firing properties from the anteroventral thalamic nucleus via the presubiculum.^50^ Although some studies show that HD cells in the dorsal presubiculum are not theta-modulated,^51,52^ others reported theta-modulated HD cells in more ventral presubiculum.^20,53^ A clarification of the circuit for theta-modulated HD cells in future experiments would be welcome.

While some parameters were set according to previous studies, several were determined heuristically (STAR Methods) to ensure that the firing characteristics of the modeled cells match those in real scenarios, such as the width of HD cell tuning, grid spacing, and the length and angle of theta sweeps. Despite the lack of experimental evidence for some of these settings, the model’s behavior—specifically, its ability to produce left-right theta sweeps—remains robust, as demonstrated by a sensitivity analysis (see Figure S7 and STAR Methods). However, certain aspects of our model warrant further investigation. First, the increased adaptation strength along the dorsal-ventral axis was measured from cells using *in vitro* patch-clamp recordings,^21^ which may not necessarily be grid cells. To confirm whether adaptation strength contributes to sweep length and angle along the dorsal-ventral axis will require *in vivo* patch-clamp recordings in behaving animals. Second, in the current model, faster running speed increases theta oscillation amplitude. In a more realistic scenario, running speed affects both the power and frequency of theta rhythmicity.^54–56^ Future modeling could explore how changes in theta frequency with running speed impact the model’s behavior. Third, while our results are based on hardwired continuous attractor networks in the HD and grid cell systems, it remains an open question whether these attractor dynamics are inherently present at birth, and if not, how they are formed during development.

In summary, we propose a simple yet comprehensive network model where left-right-alternating theta sweeps can be generated intrinsically during open-field navigation. This theoretical framework aligns with numerous experimental findings and provides testable predictions for future research. These results enhance our understanding of the neural dynamics underlying spatial navigation and memory-related cognitive functions.

## Resource Availability

### Lead contact

Further information and clarification about the code can be directed to Zi-long Ji (zilong.ji@ucl.ac.uk). Any other requests can be directed to Neil Burgess (n.burgess@ucl.ac.uk).

### Materials availability

The current study has not generated any new material.

## Star★Methods

### Key Resources Table

**Table:** 

REAGENT or RESOURCE	SOURCE	IDENTIFIER
Deposited data		
Grid cell dataset	Gardner et al.^45^	https://figshare.com/articles/dataset/Toroidal_topology_of_population_activity_in_grid_cells/16764508
Head direction cell dataset	Lomi et al.^19^	https://figshare.com/articles/datasetZ_strong_Data_code_for_Lomi_et_al_2023_strong_/22802861
Software and algorithms		
Python version 3.9	Python Software Foundation	https://www.python.org/
BrainPy	Wang et al.^57^	https://github.com/brainpy/BrainPy
replay_trajectory_classification	Denovellis et al.^58^	https://github.com/Eden-Kramer-Lab/replay_trajectory_classification
Original code and analysis	This paper	https://github.com/ZilongJi/GridCellThetaSweeps

### Experimental Model and Study Subject Details

#### The grid cell dataset

The grid cell dataset was collected by Gardner et al.^45^ from three experimentally naive male Long Evans rats (Rats 1, 2 and 3) as they foraged for randomly scattered food crumbs in a 1.5 m × 1.5 m square open-field arena. Animals’ 2D position and head direction were obtained from 3D motion capture by attaching a set of five retroreflective markers to implant during recordings. Neural recordings were obtained via Neuropixels silicon probes targeting the MEC–parasubiculum region (one recording session for Rat 1 with single-shank probes in bilateral hemispheres, one recording session for Rat 3 with multiple-shank probes in the left hemisphere, and two recording sessions for Rat 2 with single-shank probes in bilateral hemispheres). Grid cells were identified with methods described in Sargolini et al.^23^ which resulted in a total of 1330 grid cells. Specifically, for Rat 1, there were 163 grid cells from two grid modules; for Rat 2, there were 483 grid cells from three modules in session 1 and 544 grid cells from three grid modules in session 2; for Rat 3, there were 140 grid cells from one grid module. We decoded theta sweeps from Rats 1 and 2 since decoding of location was most reliable due to cells were recorded from multiple grid modules (see Figure S6 and Video S3).

#### The head direction cell dataset

The head direction cell dataset was collected by Lomi et al.^19^ from six adult male Lister Hooded rats as they foraged for randomly scattered food in a 0.9 m × 0.9 m square arena. Animals’ 2D position and head direction were obtained from video tracking of two light-emitting diodes (LEDs) on the headstage. Neural recordings were obtained via chronic recording electrodes targeting the anteroventral thalamic nucleus. Head direction cells were identified using the Rayleigh vector (passing 99^*th*^ percentile shuffle cutoff). To further identify theta modulated HD cells, Lomi et al.^19^ computed the index of rhythmicity (IR) and the index of theta phase coupling (IC) for each cell. A HD cell passing 99^*th*^ percentile shuffle cutoff for IC and having an IR ≥ 0:001 was considered as a theta × HD cell. Among these theta × HD cells, they further identified theta skipping cells with theta skipping (TS) index TS > 0.1 (see below). These operations resulted in a total of 208 HD cells, among which 76 are normal HD cells without theta modulation, 77 are theta × HD cells and 30 of them are theta skipping cells.

### Method Details

#### Details of the computational model

The computational framework consists of coupled continuous attractor networks that model theta-modulated head direction cells in the parasubiculum as a ring attractor (HD attractor) and grid cells in the MEC as a two-dimensional attractor on a neuronal sheet (GC attractor).

The HD attractor is responsible for encoding an internal direction, which can differ from the head direction of the animal, and the GC attractor maps out the animal’s location in space. Specifically, the HD cells within the HD attractor are organized in a circular arrangement corresponding to 0 to 360 degrees, with each cell firing when the animal faces a particular direction. At the population level, the center of this activity is referred to as the internal direction, which differs from the actual head direction of the animal. As the animal moves, theta rhythmic input and firing rate adaptation work together to cause the internal direction to sweep from side to side along the animal’s head axis (head direction).

This internal direction from the HD network then provides a directional input to grid cells via an intermediate layer of conjunctive grid × direction (conj-grid) cells, with a slight offset that shifts along the same direction. This directional input is represented as an external activity bump that sweeps in coordination with the internal direction in the HD network. In the GC network, the network state is characterised by a two-dimension activity bump on the attractor manifold. Due to the periodic boundary effect, when the activity bump is mapped from the toroidal manifold to physical space, it manifests as multiple bumps arranged in a hexagonal pattern. As the external input bump sweeps from side to side of the head axis at a location ahead of the current position of the animal, the activity bump in the GC attractor network exhibits left-right sweeps in a forward directed manner.

Below, we introduce each part of the model in mathematical details. For parameter settings, see Tables S1 and S2.

#### The head direction attractor network

Theta modulated HD cells are modeled as a ring attractor with the following form:

(Equation 1) $$\tau_{h}\frac{\partial h_{i}}{\partial t}=-h_{i}+\sum_{j=1}^{N_{h}}J_{ij}^{h}f_{h}\left(h_{j}\right)-a_{i}^{h}+I_{i}^{h}(V,\theta ),$$

where *h_i_* is the pre-synaptic input to *i_th_* HD cell with *i* = 1,…,*N_h_*, and *N_h_* is the number of HD cells. Each HD cell receives recurrent input from other HD cells, along with firing rate adaptation $a_{i}^{h}$ with a form of negative feedback (see below), and the head-direction dependent sensory input $I_{i}^{h}(V,\theta )$. The directional input is modeled with a Gaussian form, with the peak representing the animal’s current head direction, written as,

(Equation 2) $$I_{i}^{h}(V,\theta )=A_{h}\alpha_{h}\exp\left[\frac{{\left(x_{i}-\theta \right)}^{2}}{4b_{h}^{2}}\right],$$

where α_*h*_ represents theta modulation from medial septum with a form of sinusoidal wave (see details below). *A_h_* represents the baseline input strength of head-direction cells. *x_i_* is the preferred internal direction of the *i_th_* neurons on the ring attractor, and *b_h_* controls the width of the external Gaussian input.

*f_h_*(*h_j_*) is the firing rate of the *j_th_* HD cell. The activation function *f_h_* is modeled as a global inhibition, which is written as,

(Equation 3) $$f_{h}\left(h_{j}\right)=\frac{h_{j}^{2}}{1+k_{h}\underset{j}{\sum }h_{j}^{2}},$$

where *k_h_* represents the global inhibition strength among head-direction cells. The global inhibition is important for maintaining a localized activity bump on the ring attractor.

$J_{ij}^{h}$ represents the recurrent connections between the HD cell with preferred internal direction *i* and the HD cell with preferred internal direction *j*, with the form:

(Equation 4) $$J_{ij}^{h}=\frac{J_{0}^{h}}{2\pi b_{h}^{2}}\exp\left[\frac{||\phi_{i}-\phi_{j}|{|}_{h}^{2}}{4b_{h}^{2}}\right],$$

where ∥ *ϕ_i_* − *ϕ_j_* ∥_*h*_ denotes the circular distance of the *i_th_* HD cell with preferred internal direction *ϕ_i_* and the *j_th_* HD cell with preferred internal direction *ϕ_j_* on the ring and $J_{0}^{h}$ denote the connection strength between cells. *b_h_* controls the tuning width of HD cells.

### The grid cell attractor network

The pure grid cells are modeled as a two-dimensional continuous attractor network with the following form:

(Equation 5) $$\tau_{g}\frac{\partial g\left(\phi_{i}\right)}{\partial t}=-g\left(\phi_{i}\right)+\sum_{j}^{N_{g}}J_{ij}^{g}f_{g}\left(g\left(\phi_{j}\right)\right)-a_{i}^{g}+l_{i}^{conj\;-g}(V,\theta ),$$

where *g* (*ϕ*_*i*_)is the pre-synaptic input to *i_th_* grid cell with *i* = 1,…,*N_g_*, and *N_g_* is the number of grid cells tiling the two-dimensional neuronal sheet. *ϕ*_*i*_ = (*ϕ*_*x*_ ; *ϕ*_*y*_) with *ϕ*_*x*_ ; *ϕ*_*y*_ *∈* (0; 2π] representing the preferred phase of the *i_th_* grid cell on the attractor sheet. We set the neuronal sheet with periodic boundaries so that it forms a toroidal manifold. To account for the hexagonal firing fields of grid cells, a mapping function is applied to transform between the location in the physical space and the location in the phase space, which is written as:

(Equation 6) $$\phi =\psi (z)=\mathrm{mod}\left(W_{trans\;}z,\lambda \right)\times 2\pi ,$$

where *z*(*x*; *y*) is the coordinate of a location in the physical space, *λ* controls the grid spacing, *mod*(·,·) denotes the modulo operation and *Ψ* denotes the mapping operator. This mapping takes two steps: first, the transformation matrix *W*_*trans*_ given by:

(Equation 7) $$W_{trans\;}=\left(\begin{array}{cc} 1 & -\frac{1}{\sqrt{3}}\\ 0 & \frac{2}{\sqrt{3}} \end{array}\right),$$

projects *z* onto two axes that are sixty degrees apart; second, the modulo operation is applied to convert the transformed coordinate into a phase coordinate. This means when the animal runs into a location away from the current location, the two locations can be wrapped to similar phases in the phase space. This mapping process leads to a grid cell with six-fold symmetry in its firing pattern.^59^

Grid cells in different grid modules have different grid spacing, and can operate as independent continuous attractor networks.^45^ To model theta sweeps in different grid modules (as shown in Figure 2K and Video S2), we vary λ above to implement them.

*f_g_*(*g*(*ϕ*_*j*_))represents the firing rate of the *j_th_* grid cell, which is also modeled as a global inhibition with a form the same as in head direction cells. We denote *k_g_* as the global inhibition strength among grid cells within a module and maintain it with the same value across grid modules. $J_{ij}^{g}$ represents the recurrent connections between the grid cell with the preferred phase *ϕ* and the grid cell with *ϕ*_*j*_ the preferred phase *ϕ*_*j*_, with the form:

(Equation 8) $$J_{ij}^{g}=\frac{J_{0}^{g}}{2\pi b_{g}^{2}}\exp\left[\frac{||\phi_{i}-\phi_{j}|{|}_{g}^{2}}{4b_{g}^{2}}\right]$$

where ∥ ·∥ _*g*_ denotes the circular distance on the toroidal manifold, and $J_{0}^{g}$ denote the connection strength. *b_g_* controls the grid field width.

$I_{i}^{conj\;j-g}(V,\theta )$ represents the shifted phase input to the *i_th_* grid cell from the upstream conj-grid cells. While we didn’t explicitly model these cells in the current model (due to a high memory consumption since we need to model a 2D attractor at each direction), the shifted phase input has three important features: first, the input strength is modulated by running speed; second, it is also modulated by septal theta oscillation as the conj-grid cells are tuned to theta rhythm.^23^ Third, the direction of phase offset is aligned with the internal direction from the upstream HD attractor.^13^ Combining all these features and re-writing $I_{i}^{conj\;j-g}(V,\theta )$ as $P_{i}^{g}(\phi )$ for simplicity, the shifted phase input is expressed as:

(Equation 9) $$I_{i}^{g}(\phi )=\alpha_{g}\underset{j}{\sum }I^{conj\;-g}\left(\beta_{j}\right)=\underset{j}{\sum }f_{h}\left(h_{j}\right)T(v)\exp\left[\frac{||\phi -\psi (z)-w\left(\beta_{j}\right)|{|}^{2}}{4b_{g}^{2}}\right]$$

where *ϕ* is the phase coordinate, *α*_*g*_ represents theta modulation (see below), *I*^*conj* – *g*^(*β*_*j*_) is the input from the *j_th_* group of conjunctive grid cells with preferred internal direction as *β*_*j*_. The resulting phase input equals to a weighted sum of many 2D shifted Gaussian bumps from all the conj-grid cells with the weights as the firing rate of upstream HD cells *f_h_*(*h_j_*). *w*(*β*_*j*_) is a vector with *w*(*β*_*j*_)= *w*_0_(cos *β*_*j*_; sin *β*_*j*_), where *w*_0_ represents the offset length. This term means if the conj-grid cells coding for the direction of *β*_*j*_ are activated, they will lead to a shifted phase input with the amount of *w*_0_ along the same direction onto downstream grid cells. The strength of the resulting shifted phase input is modulated by running speed with *T*(*V*):

(Equation 10) $$T(V)=T_{0}V+A_{g}$$

where *T*_0_ and *A_g_* are both constants. This linear modulation reflects that as the animal runs faster, the firing rate of the conj-grid cells increases accordingly.^23^ Consequently, a higher speed can lead to a stronger shifted phase input, which drives the activity bump moves faster along the moving direction, and therefore potentially contribute to path integration.

### Speed modulation of theta input from the medial septum and phase input from the conj-grid cells

In our model, running speed modulates both the oscillation magnitude of medial-septal theta input and the phase input strength from conj-grid cells.^23^ Theta modulation onto HD cells is defined as:

(Equation 11) $$\alpha_{h}=1+{\bar{\alpha }}_{h}V\sin\left(\omega_{\theta }t\right),$$

where the oscillation magnitude scales linearly with the running speed *v*,^28^ with ${\bar{\alpha }}_{h}$ a scaling factor. Similarly, theta modulation onto grid cells is defined as:

(Equation 12) $$\alpha_{g}=1+{\bar{\alpha }}_{g}V\sin\left(\omega_{\theta }t\right),$$

where the oscillation magnitude also scales linearly with the running speed *v*, with ${\bar{\alpha }}_{g}$a scaling factor. While our previous modeling work^17^ has demonstrated that firing rate adaptation generates bump sweeps in a continuous attractor model of place cells, the inclusion of medial-septal theta modulation naturally constrains the bump sweep frequency to the theta rhythm, eliminating the need for precise parameter tuning. This addition enhances the model’s biological plausibility and robustness.

### Firing rate adaptation

$a_{i}^{h}$ and $a_{i}^{g}$ represent firing rate adaptation in the *i_th_* HD cell and the *i_th_* grid cell, both of which have the following form:

(Equation 13) $$\tau_{a}\frac{\partial a_{s}\left(\phi_{i}\right)}{\partial t}=-a_{s}\left(\phi_{i}\right)+m_{s}f_{s}\left(s_{i}\right),$$

where *s∈* {*h*,*g*}, and *f*_*s*_(*s_i_*) represents the firing rate of the *i_th_* cell. *τ*_*a*_ is the time constant of firing rate adaptation, which is much larger compared to *τ*_*h*_ and *τ*_*g*_, indicating that the process of firing rate adaptation is a slow dynamics compared to that of cell firing. *m*_*s*_ represents the adaptation strength, which can be different in HD cells and grid cells, as well as in different grid modules along the dorsal-ventral axis.^21^ At single neuron level, firing rate adaptation reduces the firing frequency following an initial increase in response to an input of constant intensity. At the network level, it induces intrinsic mobility of the activity bump. In fact, any forms of slow negative feedback could exhibit similar effect of increasing the intrinsic mobility, i.e., short-term depression,^15^ and hence lead to similar network-level dynamics presented in the current study.

### Parameter settings and explanation

We simulated the model proposed above using the BrainPy computational framework,^57^ with parameter settings shown in Tables S1 and S2. The simulated model consists of 100 theta-modulated HD cells (*N_h_*) and 10000 theta-modulated pure grid cells (*N_g_*) arranged on a two-dimensional neuron sheet. The rate time constants for both HD cells (*τ*_*h*_) and grid cells (*τ*_*g*_) are set to 10 ms, as is typical in rate based models (in spiking networks, this would need to be set on the order of 1ms due to the nature of spike firing).

For HD cells, the recurrent connections are modelled as Gaussian profiles with a standard deviation (*b_h_*) of 0.4 radians, which results in an average tuning width of 115° for non-rhythmic HD cells, 116° for HD cells with normal theta modulation, and 121° for HD cells with theta skipping. These values roughly match the experimental data from Lomi et.al.^19^ (96 ± 28° for non-rhythmic HD cells, 92 ± 29° for HD cells with normal theta modulation and 119 ± 12° for HD cells with theta skipping).

For grid cells, the recurrent connections are modelled as 2D Gaussian profiles with a standard deviation (*b_g_*) of 0.8, which is about 13% of the width/height of the 2D neuronal sheet. This setting primarily affects the width of each grid field, which can be further optimized by checking the grid field within empirical data. In the current study, we simply chose 0.8 as the generated field size roughly matches the real data from Gardner et.al.^45^ (averaged grid spacing from all data).

The time constant for firing rate adaptation, *τ*_*a*_, is set to a much larger value of 100 ms compared to the rate time constant *τ*_*g*_ and *τ*_*h*_. This means it takes approximately 100 ms for a neuron’s firing rate to adapt (i.e., decrease) in response to a sustained stimulus. To investigate theta sweeps along the dorsal-ventral axis where the adaptation strength increases,^21^ the adaptation strength of grid cells (*m_g_*) in our model is set as 0.7 for grid cells at the most dorsal part of the MEC and 1.8 for grid cells at the most ventral part of the MEC). Importantly, there is an inherent interdependence between the adaptation time constant and adaptation strength. While increasing the adaptation time constant reduces the rate of change in firing rate, increasing the adaptation strength counterbalances this effect. Thus, these two parameters jointly influence the negative feedback effect in generating intrinsic mobility in attractor networks.

It is noteworthy that the adaptation time constant in our model is set smaller than in previous studies, where it typically lasts from a few hundred milliseconds to several seconds. However, our choice of shorter adaptation time constant will not affect the result too much, since we can choose a smaller value of adaptation strength to match the time constant. In contrast, we can choose a longer adaptation time constant by scaling up the adaptation strength, which will lead to similar results. In future experimental studies and empirical analyses, it will be important to measure these two parameters with in-vivo whole cell recordings, and investigate their effects on theta sweeps independently.

The final important parameter is the offset value projected from conjunctive grid cells to pure grid cells, *w*_0_, which we set as 1/9 (approximately 1.8% of the width/height of the 2D neuronal sheet). While the offset has been shown in Vollan et al.,^13^ the detailed value is unclear. Further empirical data analyses will be needed to verify the actual value in future studies.

### Sensitivity analysis of model parameters

We performed a detailed sensitivity analysis of how variations in key parameters affect left-right alternation sweeps in the model. The alternation score was selected as the criterion to quantify the significance of left-right theta sweeps within the model. We investigated eight key parameters: 1&2) adaptation strength in grid and HD cells; 3&4): theta modulation amplitude in grid and HD cells; 5&6): recurrent connection noise level in grid and HD cells, i.e., the homogeneity of recurrent connections; 7) offset from conj-grid cells to pure grid cells; 8) the animal’s running speed. For each parameter, we fixed the values of all other parameters to their default settings (Table S1) and varied the parameter value in a range from 10% to 200% of the default value. The change in the alternation score is shown in Figures S7A–S7H. To further quantify the sensitivity of each parameter, we calculated the difference between the highest alternation and lowest alternation scores within the varied range, and ranked these differences for the parameters from highest to lowest (Figure S7I). We found the adaptation strength of HD cell firing has the largest effect on the model’s behavior and the theta amplitude in grid cells has the smallest effect on the model’s behavior. In general, HD cell related parameters have a larger effect on the model’s behavior than grid cell related parameters since they drive left-right sweeps of internal location in grid cells. Running speed (as expected), also has large effect on the model’s behavior since it affects theta amplitude in both HD cells and grid cells, as well as the amount of shifted phase input from conj-grid cells.

### Decoding theta sweeps in our model from the toroidal manifold to the physical space

To decode the internal location in the physical space in our model (see below for the decoding of location in real data), we take the location corresponding to the center of mass of the activity bump in the phase space of the grid cell torus and then transform it back to physical space using the inverse of the transformation matrix. While the mapping from physical location to phase location is one-to-one, the mapping from phase location to physical location is one-to-many. Therefore, to determine which physical location it maps to, we choose the one at the beginning of the simulation that is closest to the animal’s actual location. This approach reduces the one-to-many mapping to a one-to-one mapping at the start of the simulation and allows continuous one-to-one decoding from phase location to physical location until the end of the simulation. Since the phase location on the toroidal manifold does not have a boundary effect, this inverse mapping can result in a decoded position outside the current environment boundary, as reflected in Video S1 and Vollan et al.^13^

### Decoding theta sweeps in the grid cell dataset with a state-space decoder

We adopted a state-space model^58^ to decode theta sweeps in the grid cell dataset.^45^ Decoding of location was most reliable from Rat 1 (163 grid cells from two modules) and Rat 2 (544 grid cells from three modules) since cells are from multiple grid modules. The encoding part of the state-space model takes two inputs: the spike array of all spike-sorted neurons and the time-aligned 2D position data. The position data was binned into 4 × 4 cm spatial bins. We built the encoding model using a 2-ms temporal bin by up-sampling the position data from 100 Hz to 200 Hz. Only periods when the running speed exceeding 4 cm/s were considered in constructing the encoding model. Since theta sweeps closely track the animals’ physical locations, we defined latent movement dynamics as a random walk process (without considering uniform and fragmented dynamics as in their original replay analysis^58^). The movement variance for the random walk process was computed as 10 times of the movement variance estimated from the whole position trajectory during recording. This amplification helps to increase the variance of the latent movement dynamics and makes the theta sweeps more obvious (setting the amplification value to 5 or 20 did not visually change the decoding results too much).

For decoding, we used 5-fold cross-validation. In this process, we built the encoding model on 4 folds of the data and then decoded the sequences on the remaining fifth fold. This ensures that the data used for constructing a given encoding model were not used for decoding the representation. We repeated this for each fifth of the data.

### Sweep alternation score

To calculate the sweep alternation score, a three-sweep sliding window was used as in Vollan et al.^13^ Specifically, at the *i_th_* time step, a triplet of sweep directions α_*i* − 1:*i*+1_ was selected. Then the two angles between consecutive sweep pairs were calculated *a_i_* = α_*i*_ − α_*i* − 1_ and *b_i_* = α_*i*+*i*_ − α_*i*_, and the alternation score at *i_th_* time step was computed as:

(Equation 14) $$s_{i}=\frac{\left|a_{i}-b_{i}\right|}{2\cdot \max\left(\left|a_{i}\right|,\left|b_{i}\right|\right)},$$

where | ·| denotes the absolute value. The overall alternation score was finally calculated as the average of alternation scores across all simulation time bins: $s=\frac{1}{N}\cdot \sum_{i}^{N}s_{i}$, with *N* the total number of time bins. The alternation score is bound between 0 and 1, with higher values indicating more regular theta sweeps of left-right-left-right-left…

### Theta skipping index

To calculate the theta skipping index for each cell, we followed the method described in Brandon et al.^22^ First, we computed the spike time autocorrelogram (time range ± 500 ms bin width 5 ms). The autocorrelogram was further normalized by the peak value between 50 ms and 250 ms, and values above 1 (usually those around zero lag) was then clipped to 1, to ensure all the values are in a range of [0,1]. Second, the resulting autocorrelogram was fit with a cosine wave with a second interfering oscillation:

(Equation 15) $$y(x)=\left[a_{1}(\cos(\omega x)+1)+a_{2}(\cos(0.5\omega x)+1)+b\right]\times \exp\left(-\frac{\left|x\right|}{\tau_{1}}\right)+c\cdot \exp\left(-\frac{x^{2}}{2\tau_{2}^{2}}\right),$$

where *x* is the autocorrelation lag, *a*_1_ ∈ [0;1], *a*_2_ ∈ [0;1], *b*∈ [0; 1]; *c* ∈ [−1; 1]; u ∈ [10p; 18p]; t_1_ ∈ [0; 5]; t_2_ ∈ [0; 0:005] are the searching range for parameter fitting. Third, the theta skipping index was calculated as the difference between the first and second peaks in the autocorrelogram, normalized by the larger of the two:

(Equation 16) $$TS=\frac{p_{2}-p_{1}}{\max\left(p_{1},p_{2}\right)},$$

where *p*_1_ is the model value at one cycle with *x* = 2π/ω and *p*_2_ is the model value at two cycles with *x* = 4π/ω. This skipping index is bound between -1 and 1, with higher values indicating more theta skipping.

## Data Availability

The grid cell dataset is from Gardner et al.^45^ and available at: https://figshare.com/articles/dataset/Toroidal_topology_of_population_activity_in_grid_cells/16764508. The head-direction cell dataset is from Lomi et al.^19^ and available at: https://figshare.com/articles/dataset/_strong_Data_code_for_Lomi_et_al_2023_strong_/22802861. The *in vitro* patch-clamp data was requested from Yoshida et al.^21^ Code for reproducing all the results (including modeling and experimental data analysis) is available at: https://github.com/ZilongJi/GridCellThetaSweeps

## Highlights

- Shows how firing rate adaptation produces left-right theta sweeps in grid cells
- Models the interaction between internal signals for direction and location
- Explains how theta sweeps relate to theta skipping in direction cells
- Predicts theta phase coding of turning angle in head-direction cells

## In brief

Ji, Chu et al. present a network model of direction and grid cells, which generates an internal direction signal comprised of alternating left-right sweeps of encoded location and direction, based on attractor dynamics, firing rate adaptation, and theta modulation. The model explains existing data from foraging rodents and makes testable predictions.
